# National Healthcare Safety Network Antimicrobial Use Option reporting … finding the path forward

**DOI:** 10.1017/ash.2023.430

**Published:** 2023-10-23

**Authors:** Melinda M. Neuhauser, Amy K. Webb, Arjun Srinivasan

**Affiliations:** 1 Division of Healthcare Quality Promotion, Centers for Disease Control and Prevention, Atlanta, Georgia; 2 Lantana Consulting Group, Thetford, Vermont

## Abstract

Through the Centers for Medicare and Medicaid Services Promoting Interoperability Program, more hospitals will be reporting to the National Healthcare Safety Network Antimicrobial Use (AU) Option. We highlight the next steps and opportunities for measurement of AU to optimize prescribing.

The Centers for Disease Control and Prevention (CDC) Core Elements of Hospital Antibiotic Stewardship outline the importance for hospitals to monitor and benchmark antibiotic use by electronically reporting data to the National Healthcare Safety Network (NHSN) Antimicrobial Use (AU) Option.^1^ The Standardized Antimicrobial Administration Ratio (SAAR) is the risk-adjusted summary measure available to acute care hospitals participating in the NHSN AU Option. The SAAR compares observed antimicrobial days to predicted antimicrobial days for groups of antimicrobial agents used in specified patient care locations.^2–4^ Stewardship programs are using the NHSN AU Option to inform, implement, and assess interventions.^5,6^

To debate the merits of public reporting and pay for performance of SAAR data, the editors of Antimicrobial Stewardship & Healthcare Epidemiology invited a series of articles in a “pro-con” format followed by a “peacemaker” article. **Guo and Dodds Ashley** published the merits of the SAAR in the article entitled “Is the Standardized Antibiotic Administration Ratio (SAAR) ready for prime time?”,^7^ while **Shively and Morgan** published an article entitled “The CDC antimicrobial use measure is not ready for public reporting or value-based programs.”^8^ The aim of this “peacemaker” article is to provide a perspective on the path forward for AU reporting.

The SAAR was launched in 2016 as a benchmark measure of AU to meet the needs of hospital stewardship programs. Benchmarking has long been a powerful tool in quality improvement efforts as it can help facilities drive improvements based on what their peers have demonstrated to be possible. Arguably, benchmarking becomes even more useful when the goal being pursued is not a “zero” outcome like healthcare-associated infections. As **Shively and Morgan** point out, the ideal amount of AU is certainly variable based on several important contextual factors.^8^ The SAAR was designed to enable stewards to compare their hospital’s performance to others and account for the factors that might explain why higher or lower use might be justified.

**Guo and Dodds Ashley** highlight examples of the usefulness of the SAAR from the perspective of stewardship programs in hospitals around the country.^7^ They note that the SAAR has proven useful in identifying potential locations and agents where AU could be improved as well as being useful in monitoring the effectiveness of efforts to make those improvements. They state that having a measure that provides benchmarking has been helpful in engaging both providers and administrators in stewardship efforts. The fact that more than 2700 hospitals have voluntarily submitted to the NHSN AU Option demonstrates that hospitals are finding the SAAR useful in supporting their efforts to improve AU.

**Shively and Morgan** do point out several important limitations of the SAAR in its current iteration and caution against public reporting or payment based on quantitative measures of AU.^8^ The variety of patients residing in any given unit of a hospital complicates comparisons at the patient care location level using only facility-level risk adjustment. The SAAR is currently not risk-adjusted for patient-level factors, which studies suggest might improve AU risk adjustment.^9,10^

There is little debate on the need to expand access to national data on hospital AU and antimicrobial resistance (AR).^11^ A national requirement for all hospitals to report would meet that critical need. However, many options for such a requirement stipulate that the data reported must be publicly available and/or used for payment adjustments. From its outset, the CDC recognized that the SAAR was not a useful measure for either public reporting or pay for performance. Indeed, the CDC has only ever sought endorsement for the SAAR as a quality reporting and public health measure.^12^ The Centers for Medicare and Medicaid Services (CMS) Promoting Interoperability Program provides a path forward to achieve the goal of broad hospital reporting of AU (and AR) while minimizing unintended, negative consequences that public reporting and pay for performance would create.

The CMS Promoting Interoperability Program for eligible hospitals and critical access hospitals has been an important mechanism for encouraging healthcare data exchange for public health purposes through the Public Health and Clinical Data Exchange Objective. In 2022, CMS elected to use that program to enable broader availability and use of inpatient AU and AR data to guide clinical and public health action and establish a true national picture of hospital AU and the threat posed by AR. Under this new requirement, which goes into effect beginning with the electronic health record reporting period in the calendar year 2024, eligible hospitals, including critical access hospitals, must attest to being in active engagement with CDC’s NHSN to submit AU and AR data.^13^ Facilities that fail to meet this requirement or fail to claim an applicable exclusion will not receive full points for the Public Health and Clinical Data Exchange Objective.

Now that there is a requirement for hospitals to report data on AU, the stewardship field should turn its attention to the discussion on what comes next. In the short term, thousands of hospitals will need to start reporting data into the NHSN AUR Module. While there is ample experience on how this can be done, CDC is partnering with a number of organizations, including state health departments and CMS, to answer questions hospitals have and to provide direct assistance to facilitate reporting. The growing stewardship and NHSN AUR expertise in state health departments will be an important and useful resource. The CDC is also working with the Health Resources and Services Administration’s Federal Office of Rural Health Policy to support critical access hospitals in overcoming challenges of submission to the NHSN AUR Module.

The CDC is also focusing on helping stewardship programs use the NHSN AU Option and the SAAR data to drive improvements. There is nothing better to support this goal than actual experiences from a variety of hospitals that have done just that. The CDC maintains a website with examples submitted by hospitals on how they have used NHSN AU data.^14^ Building on that experience, CDC is funding the development of a comprehensive set of practical use cases for NHSN AU data.

Finally, CDC is continuing efforts to refine and expand hospital AU measurement. The CDC has already started exploring ways the SAAR could potentially be risk-adjusted with AR data, and has partnered with stewardship researchers in efforts to explore which patient-level risk factors would be most useful for SAAR risk-adjustment. That body of work, combined with the advances in electronic data reporting being made by NHSN, has made patient-level risk adjustment of the SAAR a matter of when, not if. The stewardship field also needs to consider ways to help programs measure the quality of AU, rather than just the quantity. Current methods for assessing AU quality require manual chart review, which greatly limits their application. However, the growing utility of electronic data creates new opportunities in this space. The CDC is supporting work in the Veterans Affairs Salt Lake City Health Care System and at the Universities of Michigan, Pennsylvania, and Utah to develop and test an electronic measure of the quality of prescribing for inpatient community-acquired pneumonia. Ultimately, a combination of electronic quality and quantity measures of AU will provide stewardship programs with the full suite of measurement tools to support their important work.

Measurement of hospital AU has advanced dramatically in the last decade and, come 2024, will be a CMS requirement. The articles by **Guo and Dodds Ashley** and **Shively and Morgan** both point out important opportunities to continue this journey.^7,8^
